# Biofilm-Induced Type 2 Innate Immunity in a Cystic Fibrosis Model of *Pseudomonas aeruginosa*

**DOI:** 10.3389/fcimb.2017.00274

**Published:** 2017-06-21

**Authors:** Kenny Bielen, Bart ‘S Jongers, Jan Boddaert, Tom K. Raju, Christine Lammens, Surbhi Malhotra-Kumar, Philippe G. Jorens, Herman Goossens, Samir Kumar-Singh

**Affiliations:** ^1^Molecular Pathology Group, Laboratory of Cell Biology and Histology, Faculty of Medicine and Health Sciences, University of AntwerpWilrijk, Belgium; ^2^Laboratory of Medical Microbiology—Vaccine and Infectious Disease Institute, University of AntwerpWilrijk, Belgium; ^3^Laboratory Experimental Medicine and Pediatrics, Department of Critical Care Medicine, Antwerp University Hospital and University of AntwerpEdegem, Belgium

**Keywords:** *P. aeruginosa* pneumonia, chronic pneumonia, animal model, agar beads, M2 macrophage, eosinophil, Th2, Th17

## Abstract

Biofilm-producing strains of *Pseudomonas aeruginosa* are a major cause of morbidity and mortality in cystic fibrosis (CF) patients. In these patients, increased levels of IL-17 as well as of IL-5 and IL-13 along with arginase (Arg)-positive macrophages have been observed in bronchoalveolar lavage fluid. While IL-17 is a strong proinflammatory cytokine associated with host defense against bacterial and fungal infections and is also elevated in several autoimmune diseases, IL-5/IL-13 and Arg1-positive M2 macrophages are part of the anti-inflammatory type 2 (Th2) immunity. To study whether increased IL-5 and IL-13 levels are related to biofilm formation, which is frequently observed in CF patients colonized by *P. aeruginosa*, we utilized an agarose bead-embedded *P. aeruginosa* rat model commonly employed in *in vivo* biofilm studies. We showed that “sterile” agarose bead instillation in rat notably increased lung transcript levels of IL-5 and IL-13 at two post-instillation study-points, day 1 and day 3. Concurrently, increased infiltration of type 2 innate cells such as eosinophils and Arg1 positive M2 activated macrophages (Arg1+CD68+) was also observed both at day 1 and day 3 while the proportion of M1 activated macrophages (iNOS+CD68+) at these time-points decreased. In contrast, *P. aeruginosa*-loaded beads caused a drastic elevation of proinflammatory Th1 (IFNγ, TNFα, IL-12a) and antibacterial Th17 (IL-17a, IL-17f, IL-22, IL-23a) cytokines along with a high influx of neutrophils and M1 macrophages, while Th2 cytokines (IL-5 and IL-13) drastically declined at day 1 post-infection. Interestingly, at day 3 post-infection, both Th1 and Th17 cytokines sharply declined and corroborated with decreased M1 and increased M2 macrophages. These data suggest that while IL-17 is linked to episodes of acute exacerbations of infection in CF patients, the increased Th2 cytokines and M2 macrophages observed in these patients are largely due to the biofilm matrix. The data presented here has important implications for clinical management of CF patients.

## Introduction

*Pseudomonas aeruginosa* is the major cause of pulmonary infection in cystic fibrosis (CF) patients (Gibson et al., 2003). Approximately 80% of CF patients become chronically colonized/infected with *P. aeruginosa* by late adolescence and these patients can live with such infections for years (Lyczak et al., 2002). Chronic *P. aeruginosa* colonization is also associated with increased morbidity and mortality in CF patients especially during episodes of acute exacerbations (Bhatt, 2013). This heightened vulnerability and persistence of bacterial colonization is caused by increased mucous secretion in the alveolar spaces that provide an ideal environment to form biofilms (Sadikot et al., 2005; Moreau-Marquis et al., 2008).

Biofilm mainly consists of bacterial-derived exopolysaccharides that protect the encapsulated bacteria from host immune cells and antibiotics. Interestingly, suppressive therapy reduces *P. aeruginosa* lung burden, but does not impact lung biofilm burden (Fernandez-Barat et al., 2016). To mimic this distinct pathology observed in CF and other chronic obstructive lung diseases such as bronchiectasis, agarose beads are employed to establish a chronic lung infection model. In this model, bacteria are slowly released from agarose. Agarose is a linear polymer of repeating units of agarobiose that closely mimics the extracellular polymeric substances or exopolysaccharides secreted by *P. aeruginosa* (Cash et al., 1979; Growcott et al., 2011). Genetically modified murine models based on mutations in gene encoding for cystic fibrosis transmembrane conductance regulator (CFTR) have contributed invaluably to the current understanding of CFTR function. However, the CFTR genetic models lack the development of spontaneous lung pneumonia observed in CF patients (Fisher et al., 2011). Furthermore, the bioelectric characteristics in the tracheal airways of mice and humans diverge significantly and CFTR genetic models continue to demonstrate substantial cAMP-inducible changes in chloride permeability despite the absence of a functioning CFTR (Grubb et al., 1994; Liu et al., 2006).

Chronic *P. aeruginosa* pneumonia occurring in CF patients causes a persistent lung inflammation dominated by neutrophils and an antibody response against *P. aeruginosa* (Moser et al., 2000). Neutrophils and macrophages are the most important first responders within the innate immune system. Specifically, macrophages due to their longer life-spans are the major effector population in the first line of defense against invading pathogens (Serbina et al., 2008; Hanke and Kielian, 2012). This cell type is classically divided into proinflammatory M1 macrophages that are chiefly responsible for bacterial clearance, and anti-inflammatory M2 macrophages important in tissue repair processes. M1 macrophages are commonly identified by their high inducible nitric oxide synthase (iNOS) production and M2 macrophages are identified due to their high arginase (Arg) 1 expression (reviewed in Benoit et al., 2008).

Interestingly, increased levels of anti-inflammatory Th2 cytokines (IL-5 and IL-13) and antibacterial Th17 cytokine (IL-17A) are observed in bronchoalveolar lavage (BAL) fluid of CF patients chronically colonized or infected with *P. aeruginosa* (Moser et al., 2000; Hartl et al., 2006; Tiringer et al., 2013). Several studies have also shown an increase in M2 macrophages and eosinophils in BAL fluid of CF patients who had been *P. aeruginosa* carriers at some stage in their lifetimes (Grasemann et al., 2005; Murphy et al., 2010). While CF patients frequently show a heightened Th2 and Th17 cytokine response at a given stage during the disease, it is not known whether and how these cytokines are linked to *P. aeruginosa* or its biofilm. A skewing of macrophages toward M2 polarization with increased Arg1 and reduced iNOS expression has been evidenced in catheter-associated staphylococcal biofilms (Thurlow et al., 2011). In this study, we investigated the innate immune response to biofilm-like structures in the chronic pneumonia agar bead model and showed that sterile biofilm beads could solely incite the specific type 2 immune response observed in CF patients.

## Methods

### Bacterial dose preparation

Agar beads were prepared following a previously published protocol with minor modifications (Growcott et al., 2011). Briefly, colonies from an overnight culture of *P. aeruginosa* were diluted with 30 mL sterile PBS to achieve a suspension of 0.3 O.D. at 600 nm. Cultures were washed and the pellets re-suspended in 4 mL sterile PBS. One milliliter of bacterial suspension was added to 10 mL of sterile 2% agar at 52°C and the mixture added to 15 mL heavy mineral oil (52°C) supplemented with 0.02% sorbitan-monooleate (Sigma-Aldrich) and mixed thoroughly. Beads were centrifuged at 10,000 × g at 4°C, extensively washed with sterile PBS and filtered using a 200 μm nylon mesh. Beads were resuspend in 2 × w/v PBS and 500 μL bead suspension (≈ 2E7 colony forming units) was used per animal. Sterile beads were prepared using sterile saline. Each bead solution was plated on blood-agar plates and incubated overnight at 37°C for inoculum validation.

### Anesthesia and endotracheal intubation

All animal experiments were conducted according to the guidelines of the Federation of European Laboratory Animal Science Associations and approved by the University of Antwerp Ethics Committee. Rats were anesthetized using 100 mg/kg ketamine and 1 mg/kg medetomidine and intubated utilizing a tilting intubation platform (Hallowell EMC). Briefly, anesthetized rats were placed on the tilting platform in a horizontal position. An elastic band was placed behind the front incisors and attached to the platform. Next a 14 G catheter was placed into the trachea with the aid of a guide wire. With 14 G catheter, animals were instilled with agarose beads, extubated, and anesthesia antagonized with 300 μg/kg atipamezole. Animals were placed back in their cages and followed up.

### Animal experimental groups

A total of 36 adult male Wistar rats (mean weight 340 g, *SD* = 23 g, Charles River) were used in this study and randomly assigned into one of the five experimental groups: (i) Non-manipulated animals served as the control group (*n* = 12; no bead group); (ii) Two sterile agarose (St) bead groups euthanized at day 1 and day 3 post-instillation, respectively (*n* = 6 animals per group); (iii) Two chronic pneumonia (Pa-bead) groups where animals were instilled with 2 × 10^7^ CFU of *P. aeruginosa* enmeshed in agarose beads and euthanized at day 1 and day 3 post-infection, respectively (*n* = 6 animals per group).

### Animal follow-up and euthanasia

Animals were closely monitored for clinical signs of pneumonia (SI Table 1). Euthanasia was performed by isoflurane overdose and blood collected by cardiac puncture utilizing EDTA coated tubes. The trachea was exposed and lungs lavaged using a 14-gauge angiocatheter with 20 mL/kg body-weight of ice-cold sterile PBS. Right lung was removed, washed in sterile PBS and snap frozen in liquid nitrogen. Left lung was washed and fixed overnight in 2% paraformaldehyde and prepared for paraffin embedding.

### Histopathology

Lung pathology scoring was performed on H&E stained 5 μm thick paraffin sections, as done previously (Matute-Bello et al., 2011). All lungs used for histology were treated with standardized protocols to allow comparisons between the groups. Lung pathology was assessed blinded by a trained pathologist. Amount of neutrophils for each group were manually counted on 8–10 consecutive images grabbed using 200x magnification per slide for each animal, eosinophils were quantified on 400x magnification. For slides that showed heterogenic staining pattern, most affected areas were imaged for quantification.

### Immunohistochemistry

Immunohistochemistry was done as described by us previously (Wils et al., 2012). Briefly, antigen retrieval was performed in citrate buffer (0.018 M citric acid.H_2_O and 0.082 M sodium citrate.H_2_O) using microwave heating. Endogenous peroxidase was blocked using 0.3% H_2_O_2_ for 20 min. Blocking of non-specific antigens was done using 1:5 diluted normal horse serum in 1% BSA solution in PBS (PBS-BSA) for 30 min. Primary antibodies were diluted in PBS-BSA solution and incubated overnight at 4°C: anti-CD68, 1:200 dilution (Abd Serotec MCA341R); anti-arginase-1, 1:400 dilution (Santa Cruz Sc-18354); and anti-iNOS, 1:100 (Abcam ab15323). Secondary biotinylated antibodies (Jackson Immunoresearch) were used in 1:200 dilution, incubated for 30 min at room temperature, washed and incubated with extravidin-HRP for 30 min followed by DAB (5', 5' diaminobenzedine, Dako) development. Double-labeled immunohistochemistry was performed using anti-CD68 (1:200 dilution), anti-arginase1 (1:400 dilution), and anti-iNOS (1:100 dilution) with DAG-cy3, DAM-cy5 and DAR-cy5 in 1:200 dilutions. Sections were co-stained with 5 μg/mL DAPI (Sigma-Aldrich) and coverslipped using antifading PBS-glycerol mounting medium (Citifluor).

Light microscopy images were grabbed on Olympus UC30 color camera. Quantification was performed by calculating the percentage of stained area using pixel-by pixel analysis after spectral de-convolution of the image using the IHC profiler plug-in in ImageJ v1.47 (Varghese et al., 2014). The mean percentage of positive stained area per animal was used. Slides were randomized before analyses by a blinded investigator. High-resolution images from double-labeled immunofluorescence stained sections were grabbed using a dual spinning disk confocal microscope (Ultra*View* VoX, PerkinElmer) and images analyzed using Volocity (PerkinElmer). Proportions of Arg1+CD68+ and iNOS+CD68+ cells were manually counted by the blinded investigator.

### Lung transcript analyses

Total RNA from lung tissue was extracted using RNeasy-mini spin columns (Qiagen) after grinding in liquid nitrogen. RNA integrity and concentrations were estimated using RNA-nanochips on Bio-analyzer (Agilent). Extracted RNA was converted to cDNA by reverse transcriptase (RT^2^ First Strand Kit, Qiagen). Quantitative PCR was performed using CFX connect (Bio-Rad) using custom PCR-arrays (Qiagen) for following genes of interest: *IL-1*β*, IL-4, IL-5, IL-6, IL-10, IL-12a, IL-13, IL-17a, IL-17f, IL-22, IL-23a, Ifn*γ*, Tnf*α. Housekeeping genes included were: *Ywhag, Actb, Polr2b, Gapdh, Sdha*, and *Tbp*. Additionally, a random genomic DNA contamination control, three reverse transcriptase controls, and three additional PCR controls were used. Data was validated on independently extracted RNA and analyzed using in-house primers (primer sequences available on request) using SYBRGreen assay. Data was analyzed using comparative C_T_ method as described earlier (Kumar-Singh et al., 2006; Schmittgen and Livak, 2008). Briefly, the combined average Ct value of the control group was used to calculate the fold differences in the study groups.

### Data analyses and statistics

Data analyses were performed using SPSS version 23 (IBM) and presented as either averages or average fold-differences with the standard errors of mean. Kolmogorov-Smirnov test for normality was used for each data set before testing statistical significance of differences on log-transformed values by one-way ANOVA with *post-hoc* Bonferroni corrections. Survival analyses were performed using Kaplan Meier estimator with Mantel-Cox log rank test for testing significant differences. Values of all significant correlations (*P* < 0.05) are given with degree of significance indicated (^*^P < 0.05, ^**^P < 0.01, ^***^P < 0.001).

## Results

### Sterile biofilm matrix-mimicking agar beads induce Th2 cytokine expression

**Figure 1:**
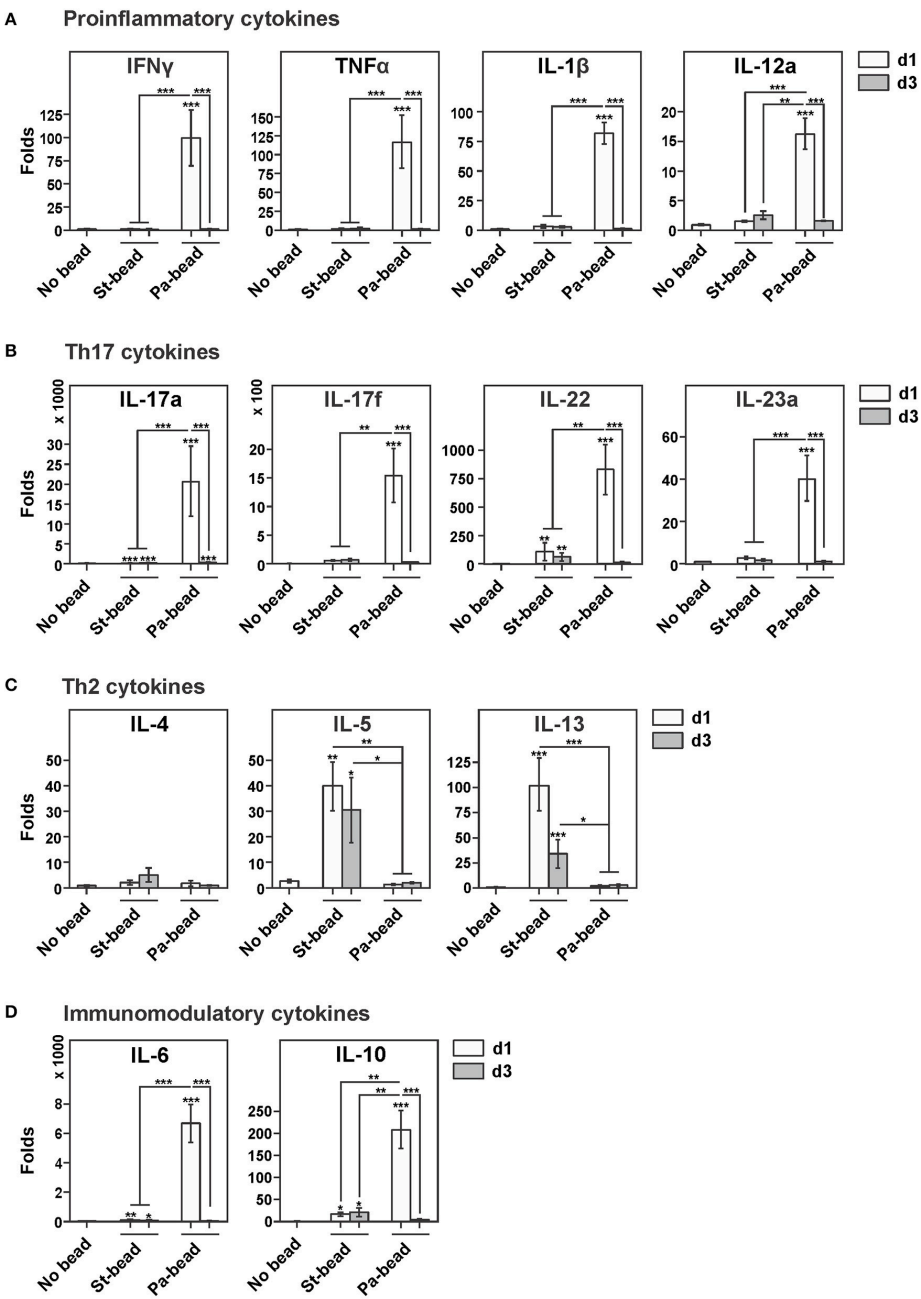
Lung transcript levels of key cytokines in response to intratracheal instillation in rats of sterile agar beads or *P. aeruginosa*-loaded beads **(A)**. Lung transcript levels of main proinflammatory cytokines *Ifn*γ, *Tnf*α, *IL-1*β, and *IL-12a* for sterile beads (St-bead) and for *P. aeruginosa* beads (Pa-bead) at post-infection time-points day (d)1 (white columns) and d3 (gray columns), compared to control animals (No bead). The most drastic elevation for these cytokines was noted for Pa-bead group at d1. **(B)** Similarly, Th17 cytokine analysis of *IL-17a, IL-17f, IL22, IL23a* showed highest lung expression levels in Pa-bead group at d1 post-infection. **(C)** Increased expression of Th2 cytokines *IL-5* and *IL-13* was noted for St-bead group at both d1 and d3 time-points. **(D)** Highest lung expression levels of immunomodulatory cytokines *IL-6* and *IL-10* for Pa-bead group at d1. **(A–D)**, ^*^P < 0.05; ^**^P < 0.01; ^***^P < 0.001; asterisk above the vertical bars denotes significance against No bead control group; *n* = 6 animals per group.

To study whether a Th2 response could be provoked by the presence of biofilm matrix, we intratracheally instilled animals with sterile agar beads and analyzed lung transcript levels of key cytokines at day 1 (d1) and day 3 (d3) post-bead instillation time points. Compared to non-manipulated control animals (No bead group), sterile beads did not cause an increase in proinflammatory Th1 cytokine expression of *Ifn*γ*, Tnf*α, *IL-1*β, IL-12a at d1 post-bead instillation (Figure 1A, white bars). Analyzing the lung transcript levels for the Th17 cytokine family, we showed increased expression of *IL-17a* (157-fold, *P* < 0.001) and *IL-22* (112-fold, *P* < 0.01) at d1 post-bead instillation that were sustained at the d3 time-point (*IL-17a*, 158-fold, *P* < 0.001; *IL-22*, 65-fold, *P* < 0.01; Figure 1B).

Interestingly, lung transcript levels of Th2 cytokines in sterile agar beads-instilled animals were markedly elevated with increased expression of *IL-5* and *IL-13* at d1 post-bead instillation (*IL-5*, 39-fold, *P* < 0.01; *IL-13*, 102-fold, *P* < 0.001) that remained elevated at d3 (*IL-5*, 30-fold, *P* < 0.05; *IL-13*, 26-fold, *P* < 0.001). Transcript levels of *IL-4* were not altered by sterile agar-bead instillation (Figure 1C). Additionally, elevated transcript levels compared to control group were also noticed for immunomodulatory cytokines *IL-6* (d1, 38-fold, *P* < 0.001; d3, 21-fold, *P* < 0.05) and *IL-10* (d1, 15-fold, *P* < 0.05; d3, 20-fold, *P* < 0.05; Figure 1D).

### Th2 innate cellular pulmonary infiltration induced by “sterile” agar bead instillation

**Figure 2:**
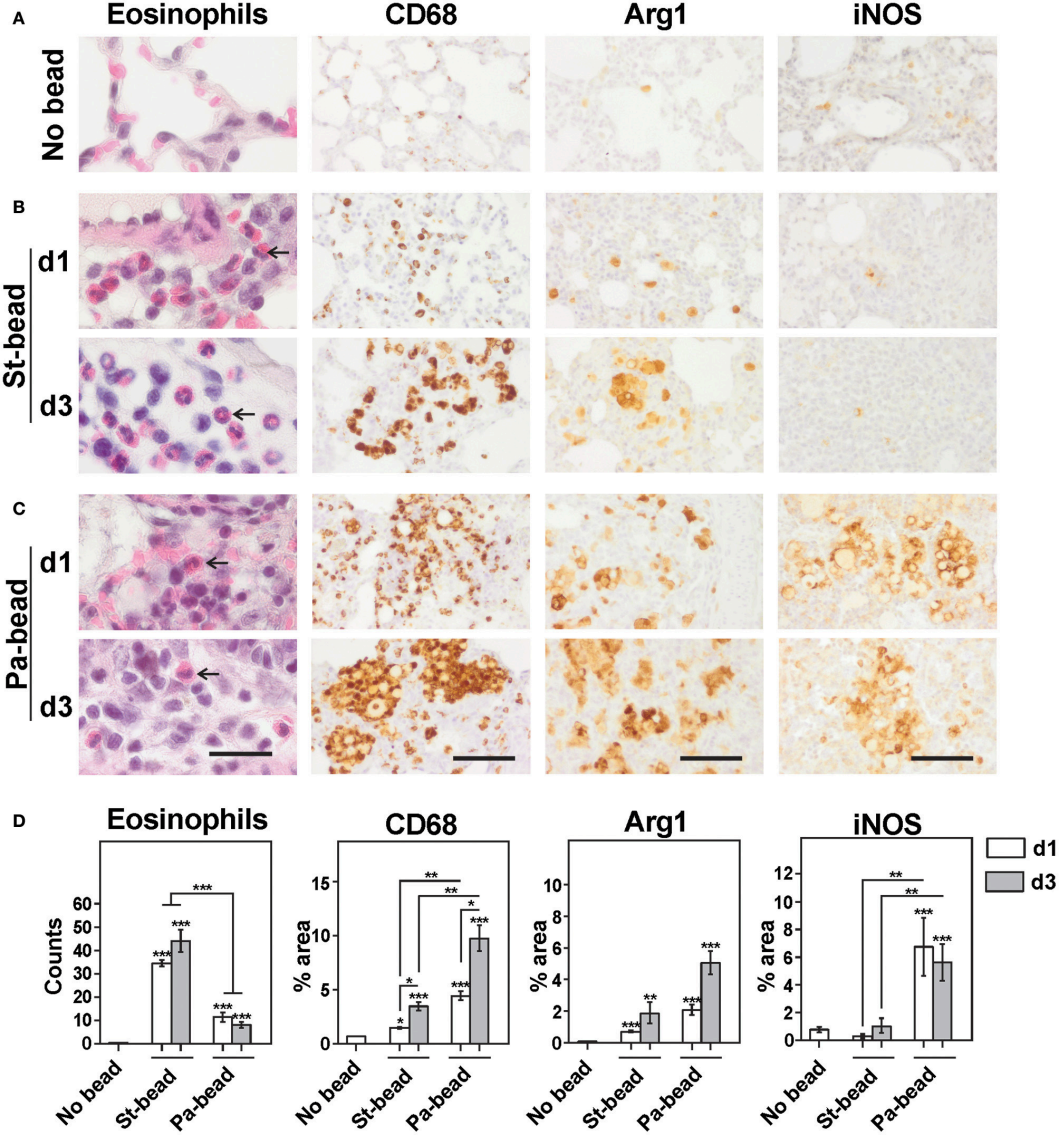
Cellular infiltrates in sterile beads and CF rat model. Representative images for H&E and immunohistochemistry for CD68, Arg1 and iNOS for **(A)** No bead control group, **(B)** Sterile bead group (St-bead) and **(C)** *P. aeruginosa* bead group (Pa-bead). An increased influx of eosinophils was observed in St-bead group but also to a limited extent in the Pa-bead group (arrows). **(D)** Quantitative analyses for the histochemical and immunohistochemical stains. Data in **(D)** is presented as averages ± SEM; ^*^P < 0.05, ^**^P < 0.01, ^***^ *P* < 0.001, and asterisk above the vertical bars denotes significance against No bead control group; *n* = 6 animals per group. **(A–C)**, scale bar represents 20 μm for H&E stained and 60 μm for immunostained sections.

IL-5 and IL-13 are potent inducers of eosinophil chemotaxis in the lung (Pope et al., 2001) and IL-13 has been shown to be involved with M2 macrophage polarization causing depressed antibacterial immunity (Knippenberg et al., 2015). Therefore, we next studied these two innate type 2 effector cells. Compared to control animals, sterile beads caused a moderate increase in neutrophil infiltration in lung at both time-points (*P* < 0.05 for both), confirming previous observations (Cash et al., 1979; Growcott et al., 2011) (SI Figure 1). Additionally, sterile beads also caused an intense eosinophilic infiltration at both studied time-points (*P* < 0.001 for both; Figures 2A,B,D), especially in regions surrounding bronchioles harboring agarose beads.

**Figure 3:**
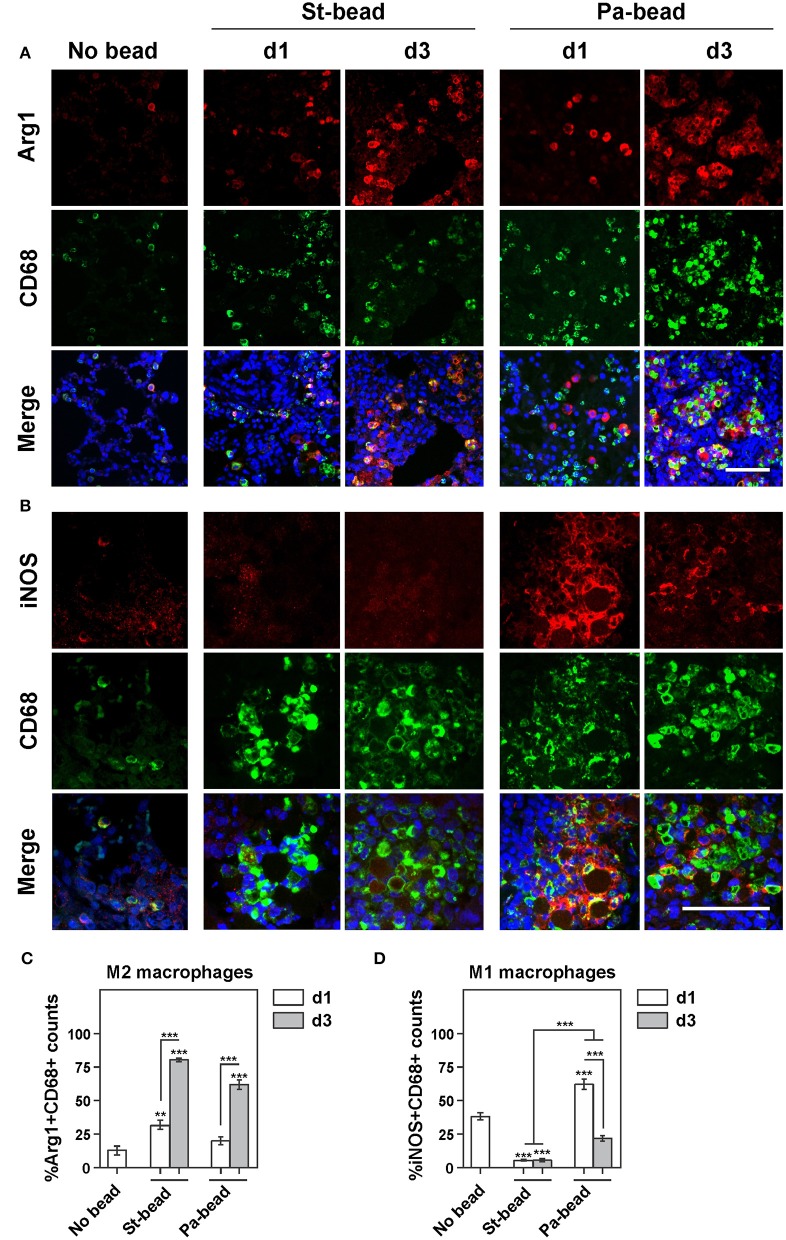
Increased M2 macrophage infiltration following bead instillation **(A)**. Representative images of double-labeled immunohistochemistry for Arg1+CD68+ cells (M2 macrophages) for control (No bead), sterile beads (St-bead), and *P. aeruginosa* beads (Pa-bead) group. **(B)** Representative images of double-labeled immunohistochemistry for iNOS+CD68+ cells (M1 macrophages) for these three groups. Quantitative analyses of **(C)** M2 cells (Arg1+CD68+) and **(D)** M1 cells (iNOS+CD68+) presented as average percentage of total CD68+ cell population ± SEM; ^**^P < 0.01, ^***^P < 0.001. Asterisk above the vertical bars denotes significance against No bead control group. *n* = 6 animals per group. Scale bars in **(A,B)** represents 30 μm (iNOS immunoreactivity was assessed at a higher magnification).

Similarly, an intense macrophage infiltration was also associated with sterile beads. However, due to overlapping macrophages especially around the agarose beads, an automated quantification estimating percentage area occupation of immunohistochemical staining was employed as described in *Methods*. With this protocol, we showed that CD68+ areas were significantly increased at d1 and d3 (two and five times, respectively), compared to control animals (*P* at least < 0.05 for both; Figures 2A,B,D). This coincided with significantly increased Arg1+ areas at both time-points (*P* at least < 0.01; Figures 2B,D). These data were confirmed on confocal double-labeled immunofluorescence microscopy showing that 31% of activated CD68+ macrophages were Arg1+ already at d1 and this proportion of M2 macrophages increased to 82% at d3 compared to controls (*P* < 0.01 for d1; and *P* < 0.001 for d3 group; Figures 3A,C). Additionally, a proportional drop in numbers of M1 macrophages (iNOS+CD68+) was observed compared to controls (*P* < 0.001; Figures 3B,D). These data indicate that agarose biofilm structures induce an acute Th2 innate cellular pathology marked by elevated eosinophils and M2 macrophages in rat lung.

### High Th17 cytokine profile in a *P. aeruginosa* agar bead pneumonia model

To study how relevant agarose/biofilm-induced Th2/Th17 cytokines are compared to the infectious situation, and whether these Th2 cytokines and Th2 innate cells observed in the sterile bead model persist after *P. aeruginosa* co-challenge, we established a Pa-bead model. In this model, rats were intratracheally instilled with agarose beads enmeshed with *P. aeruginosa* that led to development of progressive pneumonia evident up to d3 post-infection (SI Figure 2A). Pa-bead animals showed ≈50% mortality at d3 post-infection whereas sterile bead animals showed no mortality (Kaplan-Meier, ^***^P < 0.001, Mantel-Cox log rank test; SI Figure 2B).

Intratracheal instillation of *P. aeruginosa* enmeshed beads caused a drastic reduction in the observed Th2 response with both *IL-5* and *IL-13* attenuated by up to 20 and 30 times, respectively, compared to sterile bead instilled animals (Figure 1C). This coincided with an expected surge at d1 of all studied proinflammatory cytokines (*Ifn*γ, 99-fold; *Tnf*α, 117-fold; *IL-1*β, 82-fold; *IL-12a*, 16-fold; *P* for all < 0.001; Figure 1A). In accordance with earlier studies (Lavoie et al., 2011; Lovewell et al., 2014), a high expression of immunomodulatory cytokines *IL-6* (6,600-fold) and *IL-10* (211-fold; *P* for both < 0.001) was also observed (Figure 1D).

Interestingly, the surge in IL-17 family of cytokines was much more pronounced. At d1 post-infection, *IL-17a* and *IL-17f* were upregulated 25,000 and 15,000-fold, respectively, compared to controls, and ≈20,000 and ≈1,500-fold compared to sterile bead-instilled animals (Figure 1B). These cytokines are not only important in mediating neutrophil chemotaxis (Xu et al., 2014), but are also amongst the important drivers of proinflammatory cytokine expression including *IL-1*β and *Tnf*α that we also observed to be elevated in our Pa-bead model. IL-23, another member of the IL-17 family and one of the strongest drivers of Th17 cell differentiation and IL-17 secretion (Bedoya et al., 2013) was also significantly elevated (Figure 1B). Lastly, IL-22 that along with IL-17 has important functions in mucosal immunity against extracellular pathogens, particularly Gram-negative organisms (Aujla et al., 2008; Xu et al., 2014) was also elevated by several 100-folds in lung transcripts (Figure 1B). However, at d3 post-infection, transcripts of the Th17 family subsided to levels comparable to sterile bead-instilled animals. These data suggest that the IL-17 response observed in CF patients is primarily pathogen-driven.

### Increased Arg1+ activated M2 macrophages and eosinophils in *P. aeruginosa* agar bead pneumonia model

While patient studies have shown an increased arginase activity in BAL fluid of CF patients colonized with *P. aeruginosa* (Grasemann et al., 2005; Murphy et al., 2010), the individual contribution of the pathogen and the biofilm matrix remain unknown. Additionally, as bacteria are one of the most important drivers of M1 macrophage polarization, we studied whether the increased type 2 innate cellular infiltrates induced by sterile agarose beads persist in the Pa-bead model.

At both d1 and d3 time-points, Pa-bead-instilled animals were characterized by high loads of neutrophils compared to controls (*P* < 0.01; SI Figure 1) and to the sterile-bead group (*P* < 0.01 for d1; *P* < 0.05 for d3; SI Figure 1), as shown previously (van Heeckeren and Schluchter, 2002; Growcott et al., 2011). Interestingly, while eosinophilic counts in the Pa-bead group were reduced at d1 and d3 time points (≈3 and ≈5 times, respectively), these were still highly elevated in the Pa-bead group compared to the controls (*P* < 0.001; Figures 2C,D). These data are also consistent with sporadic reports of increased eosinophil activation in lung of CF patients with *P. aeruginosa* pneumonia (Koller et al., 1994).

Importantly, we showed that the Pa-bead group had ≈3-fold increased lung infiltration of CD68+ cells at both d1 and d3 time-points compared to the sterile bead group (*P* < 0.001; Figures 2C,D). Studying the M1/M2 macrophage markers, we further showed that iNOS+ cells were drastically elevated in the Pa-bead group especially at d1 (d1, 19 times and d3, 5 times, *P* < 0.01 for both), while Arg1+ cells showed a non-significant increase in infiltration in the Pa-bead model at both time-points compared to sterile bead-inoculated animals (≈3 times for both; Figures 2C,D). Double-labeled immunofluorescence microscopy confirmed these data showing that at d1, 65% of CD68+ activated macrophages were iNOS+ M1 macrophages (*P* < 0.001; Figures 3B,D), while the population of Arg1+ M2 macrophages was 18% and non-significantly elevated compared to controls (Figures 3B,C). Moreover, at d3, the Arg1+ M2 macrophage proportion increased to 63% of the total activated macrophage population (*P* < 0.001; Figures 3B,C) while iNOS+ M1 macrophages declined to 18% (*P* < 0.001; Figures 3B,D). These data indicate that chronic *P. aeruginosa* infection can sustain the anti-inflammatory M2 cellular innate immune response induced by biofilm matrix-mimicking agarose bead-instillation.

## Discussion

We showed here that instillation of “sterile” agarose beads in a rat biofilm model, extensively used in the field of cystic fibrosis research, activates the same Th2 cytokines (IL-5 and IL-13) and Th2 innate cells (eosinophils and Arg1+ M2 macrophages) as observed in human CF patients (Moser et al., 2000; Grasemann et al., 2005; Murphy et al., 2010; Tiringer et al., 2013). Interestingly, co-challenge with *P. aeruginosa* caused an acute drastic elevation of Th1/Th17 cytokine transcripts in our model, however, expression of these cytokines declined rapidly by d3. While the precise reason for this drastic decline is not known, it should be noted that animals with the most severe pathology did not survive up to the d3 time-point and this could in part have an influence here. Nevertheless, a decline in Th1/Th17 at d3 corroborated with a drastic drop in M1 macrophages in lungs with a notable increase in the Arg1+CD68+ M2 macrophage population with ≈60% of all activated macrophages being of the M2 phenotype by this time point. Although the upstream driver of this M2 polarization is so far undefined, our data suggest that the Th2/M2 response observed in CF patients is primarily due to biofilm matrix structures rather than a direct effect of the accompanying pathogen. This suggestion also fits well with the observation that pathogens most frequently associated with CF or with other chronic pneumonia pathologies, such as *P. aeruginosa, S. aureus*, and *B. cepacia* (Coutinho et al., 2008), have a high biofilm producing capacity.

Similar to human CF patients showing increased IL-5 and IL-13 but not IL-4 levels (Hauber et al., 2003; Tiringer et al., 2013), we also observed only a non-significant increase in IL-4. While IL4 is a key Th2 cytokine, eosinophil-specific IL-13 expression, but not IL-4, has been identified as a major factor for inducing allergic Th2 pulmonary pathologies (Jacobsen et al., 2015). Moreover, increased M2 macrophage infiltration in lung is observed in IL-13-associated lung diseases (Wu et al., 2015) and IL-13 has been shown to independently enhance the M2 macrophage phenotype *in vitro* (Doyle et al., 1994). Similarly, IL-5 on its own is also one of the major chemoattractants for eosinophils (Pope et al., 2001), that, together with M2 macrophages, constitute the two major innate Th2-type effector cells. Interestingly, we also showed in our model a high expression of IL-10, which similar to IL-13 can directly enhance the M2 macrophage phenotype (Makita et al., 2015).

Important for therapeutic implications, and more central to our findings here, increased expression of IL-13 and IL-5 in lungs of CF patients is associated with occurrence of acute pneumonia exacerbations (Tiringer et al., 2013). Moreover, IL-13 induced M2 macrophages can cause reduced antibacterial immunity (Knippenberg et al., 2015). Recent data also suggest that development of acute exacerbations of infection is not caused by the acquisition of new strains but rather a clonal expansion of existing strains Stenbit and Flume, 2011). Perhaps, the development of an anti-inflammatory Th2/M2 environment in the lung with lowered anti-bacterial Th1/M1 immunity in response to biofilm, that we describe here, results in a local immunosuppressive milieu that eventually aids bacterial survival and dispersal. Worryingly, one fourth of all CF patients developing an acute exacerbation do not recover to baseline within 3 months after starting treatment with antibiotics, especially those antibiotics that are linked with increased biofilm formation (Hoffman et al., 2005; Bhatt, 2013). Our data thus indicate that the upregulated Th2/M2 pathway identified here could be a potential drug target used as an antibiotic adjuvant to treat episodes of acute exacerbations in CF patients. In addition, high IL-13 and IL-5 expression could also be used to detect patients at risk of developing acute exacerbations of infection.

We also showed here in a well-described animal model of CF that the acute anti-*P. aeruginosa* immune responses are especially modulated by the IL-17 family of cytokines, members of which are IL-17A, IL-17F, IL-22, and IL-23. High lung transcript levels of all of these cytokines along with an intense migration in the lung of neutrophils and of iNOS+CD68+ M1 macrophages were observed as a direct response toward the pathogen. Although the IL-17 cytokine family is associated with cells of the adaptive arm of the immune system, in bacterial infections such as those caused by *P. aeruginosa*, innate immune cells are the first responders in secretion of IL-17, IL-21, IL-22, and IL-23 (Nieuwenhuis et al., 2002; Aujla et al., 2008; Coquet et al., 2008; Sutton et al., 2012). In this respect, both NKT cells and γδ-T cells have been identified as major sources of IL-17 production at mucosal surfaces, providing protection against *P. aeruginosa* (Nieuwenhuis et al., 2002; Coquet et al., 2008; Sutton et al., 2012), and NK cells have been shown to secrete IL-22 following Gram-negative pneumonia (Aujla et al., 2008). However, the observed antibacterial response mediated by IL-17 family of cytokines was transient and diminished rapidly. As discussed, the majority of activated macrophages were of the M2 phenotype at d3, indicating the initiation of a healing response or an anti-inflammatory response likely mediated by the biofilm-matrix. We stipulate that the precise timing for this switch in the CF agarose model would depend on the absolute and relative amounts of *P. aeruginosa* and agarose matrix challenge.

To conclude, we showed here that the Th2-mediated anti-inflammatory milieu observed in CF patients is primarily driven by the biofilm matrix structures and not by the accompanying pathogen, i.e., *P. aeruginosa*. These data prompt considering the use of IL-5 and IL-13 as biomarkers to detect and treat impending infections in CF patients. We also showed that the acute IL-17 superexpression is directly linked to episodes of acute exacerbations of *P. aeruginosa* infection in rat. These data, we believe, have important implications for the clinical management of CF patients.

## Author contributions

SK designed and supervised the study. KB, B‘S, JB, and CL carried out the experiments and analyzed data. SK performed the pathological analysis. KB, PJ, SM, HG, and SK interpreted the data. KB and SK wrote the manuscript. All authors read, edited, and approved the final draft.

### Conflict of interest statement

The authors declare that the research was conducted in the absence of any commercial or financial relationships that could be construed as a potential conflict of interest.
