# Supplementary material for: Biofilm-Induced Type 2 Innate Immunity in a Cystic Fibrosis Model of Pseudomonas aeruginosa
Source: Front Cell Infect Microbiol. 2017 Jun 21;7:274. doi: 10.3389/fcimb.2017.00274 (PMC5478716; doi:10.3389/fcimb.2017.00274)
Supplement: Supplementary file 1 [file Image1.pdf]

frontiers

Supplementary Material

Biofilm-induced type 2 innate immunity in a cystic fibrosis model of *Pseudomonas aeruginosa*

Kenny Bielen[1,2], Bart 'S Jongers[1], Jan Boddaert[1], Tom K Raju[1], Christine Lammens[2], Surbhi Malhotra-Kumar[2], Philippe G Jorens[3], Herman Goossens[2], Samir Kumar-Singh[1,2*]

***Correspondence:**

Corresponding author mailing address: Prof. dr. S. Kumar-Singh, MD, PhD, Molecular Pathology Group, Laboratory of Cell Biology and Histology, Faculty of Medicine and Health Sciences – D.T.1.32, University of Antwerp, Universiteitsplein1, B-2610 Wilrijk, Belgium. Phone: 32-3-265-3329. E-mail: samir.kumarsingh@uantwerpen.be

SI Figure 1. Neutrophil infiltration in sterile bead and Pa-bead instilled animals

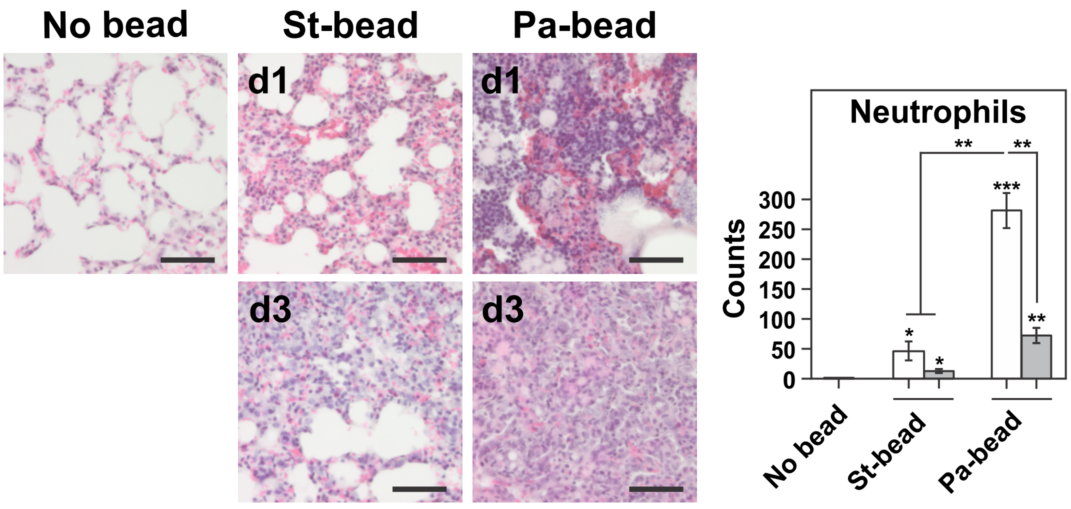

Representative images for H&E stained sections showing neutrophil infiltration for control animals (No bead), sterile agar (St-) bead instilled animals and *P. aeruginosa* loaded (Pa-) bead instilled animals showing increased influx of neutrophils on H&E stain. Scale bar represents 60 µm. Data is presented as averages ± SEM, n = 4 - 6 animals per group. Asterisk above the vertical bars denotes significance against No bead control group.
