# Supplementary material for: Biofilm-Induced Type 2 Innate Immunity in a Cystic Fibrosis Model of Pseudomonas aeruginosa
Source: Front Cell Infect Microbiol. 2017 Jun 21;7:274. doi: 10.3389/fcimb.2017.00274 (PMC5478716; doi:10.3389/fcimb.2017.00274)
Supplement: Supplementary file 2 [file Image2.pdf]

frontiers

Supplementary Material

Biofilm-induced type 2 innate immunity in a cystic fibrosis model of *Pseudomonas aeruginosa*

Kenny Bielen[1,2], Bart 'S Jongers[1], Jan Boddaert[1], Tom K Raju[1], Christine Lammens[2], Surbhi Malhotra-Kumar[2], Philippe G Jorens[3], Herman Goossens[2], Samir Kumar-Singh[1,2*]

***Correspondence:**

Corresponding author mailing address: Prof. dr. S. Kumar-Singh, MD, PhD, Molecular Pathology Group, Laboratory of Cell Biology and Histology, Faculty of Medicine and Health Sciences – D.T.1.32, University of Antwerp, Universiteitsplein1, B-2610 Wilrijk, Belgium. Phone: 32-3-265-3329. E-mail: samir.kumarsingh@uantwerpen.be

SI Figure 2. Survival and clinical follow up for sterile bead and *P. aeruginosa* loaded bead instilled animals

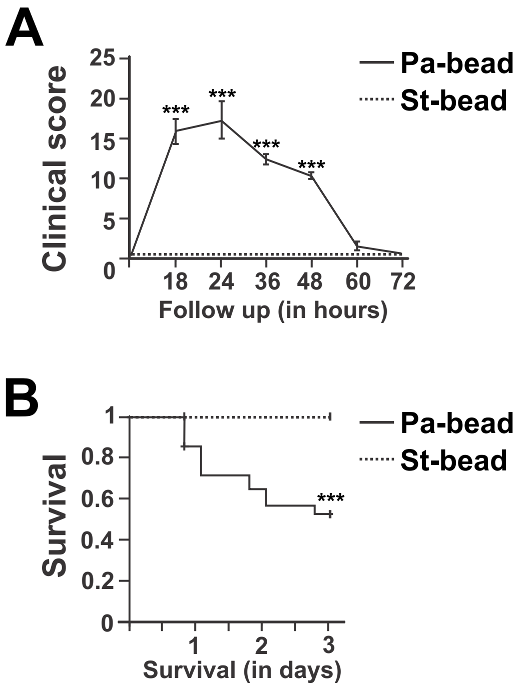

(**A**) *P. aeruginosa* (Pa)-bead model showed gradual disease progression measured using scoring scheme shown in SI Table 1 (****P* < 0.001).
(**B**) Pa-bead model showed higher mortality (Kaplan-Meier, ****P* < 0.001, Mantel-Cox log rank test) compared to sterile (St) bead instilled animals**.**
A–B Data from 67 animals (n = 52 for Pa-bead, n = 15 for St-bead groups) was available to estimate disease progression and survival.
