# Supplementary material for: Biofilm-Induced Type 2 Innate Immunity in a Cystic Fibrosis Model of Pseudomonas aeruginosa
Source: Front Cell Infect Microbiol. 2017 Jun 21;7:274. doi: 10.3389/fcimb.2017.00274 (PMC5478716; doi:10.3389/fcimb.2017.00274)
Supplement: Supplementary file 3 [file Table1.docx]

Supplementary Material

**Biofilm-induced type 2 innate immunity in a cystic fibrosis model of *Pseudomonas aeruginosa***

**Kenny Bielen^1,2^, Bart ‘S Jongers^1^, Jan Boddaert^1^, Tom K Raju^1^, Christine Lammens^2^, Surbhi Malhotra-Kumar^2^, Philippe G Jorens^3^, Herman Goossens^2^, Samir Kumar-Singh^1,2*^**

***Correspondence:**

Corresponding author mailing address: Prof. dr. S. Kumar-Singh, MD, PhD, Molecular Pathology Group, Laboratory of Cell Biology and Histology, Faculty of Medicine and Health Sciences – D.T.1.32, University of Antwerp, Universiteitsplein1, B-2610 Wilrijk, Belgium. Phone: 32-3-265-3329. E-mail: samir.kumarsingh@uantwerpen.be

**Supplementary Table 1.** Clinical scoring scheme used for monitoring disease progression in pneumonia models where animals can have a maximum score of 28 points.

| **Stage disease development** | **Description of clinical symptoms** | **Points** |
| --- | --- | --- |
| **Stage 1: early signs of pneumonia** | Avoidance | 1 |
|  | Loss of appetite | 1 |
|  | Fever | 1 |
|  | Piloerection | 1 |
|  | Rapid breathing | 1 |
|  | Ruffled fur | 1 |
| **Stage 2: signs of severe inflammation and infection** | Labored breathing | 2 |
|  | Hunched posture | 2 |
|  | Wheezing, small cough | 2 |
|  | Lethargy | 2 |
|  | Periorbital congestion | 2 |
| **Stage 3: signs of compromised lung function** | Very shallow breathing | 3 |
|  | Prostrate, not reactive | 3 |
|  | Gasping | 3 |
|  | Cyanosis at the tip of tail and feet | 3 |
